# The *Fg*Not3 Subunit of the Ccr4-Not Complex Regulates Vegetative Growth, Sporulation, and Virulence in *Fusarium graminearum*

**DOI:** 10.1371/journal.pone.0147481

**Published:** 2016-01-22

**Authors:** Duc-Cuong Bui, Hokyoung Son, Ji Young Shin, Jin-Cheol Kim, Hun Kim, Gyung Ja Choi, Yin-Won Lee

**Affiliations:** 1 Department of Agricultural Biotechnology and Center for Fungal Pathogenesis, Seoul National University, Seoul, Republic of Korea; 2 Center for Food and Bioconvergence, Seoul National University, Seoul, Republic of Korea; 3 Division of Applied Bioscience and Biotechnology, Institute of Environmentally Friendly Agriculture, College of Agriculture and Life Sciences, Chonnam National University, Gwangju, Republic of Korea; 4 Eco-friendly New Materials Research Group, Research Center for Biobased Chemistry, Division of Convergence Chemistry, Korea Research Institute of Chemical Technology, Daejeon, Republic of Korea; The University of Wisconsin - Madison, UNITED STATES

## Abstract

The Ccr4-Not complex is evolutionarily conserved and important for multiple cellular functions in eukaryotic cells. In this study, the biological roles of the *Fg*Not3 subunit of this complex were investigated in the plant pathogenic fungus *Fusarium graminearum*. Deletion of *FgNOT3* resulted in retarded vegetative growth, retarded spore germination, swollen hyphae, and hyper-branching. The Δ*Fgnot3* mutants also showed impaired sexual and asexual sporulation, decreased virulence, and reduced expression of genes related to conidiogenesis. *Fgnot3* deletion mutants were sensitive to thermal stress, whereas *NOT3* orthologs in other model eukaryotes are known to be required for cell wall integrity. We found that *Fg*Not3 functions as a negative regulator of the production of secondary metabolites, including trichothecenes and zearalenone. Further functional characterization of other components of the Not module of the Ccr4-Not complex demonstrated that the module is conserved. Each subunit primarily functions within the context of a complex and might have distinct roles outside of the complex in *F*. *graminearum*. This is the first study to functionally characterize the Not module in filamentous fungi and provides novel insights into signal transduction pathways in fungal development.

## Introduction

*Fusarium graminearum* is an ascomycetous fungus that causes *Fusarium* head blight in cereal crops worldwide, including wheat, barley, and rice, as well as ear and stalk rot in maize [1, 2]. Fungal infection of *F*. *graminearum* leads to yield and quality losses as well as contamination of grains by the production of mycotoxins (trichothecenes and zearalenone) that threaten human and animal health [3]. *F*. *graminearum* produces both sexual (ascospores) and asexual (conidia) spores [4]. Ascospores are produced and discharged from the perithecia during flowering and function as primary inocula [2, 5]. The initial structures or associated hyphae of the perithecia also serve as survival structures for overwintering [2, 5]. Conidia are responsible for secondary infections that are produced from sporodochia present on infected crops [5]. The biological processes of sexual and asexual sporulation in *F*. *graminearum* are under precise temporal and spatial regulation related to various cellular processes [6–11].

The Ccr4-Not complex is an evolutionarily conserved multi-subunit complex required for numerous cellular processes [12]. Decades of studies on model eukaryotes have revealed that the Ccr4-Not complex regulates multiple nuclear and cytoplasmic steps in gene expression, such as transcription initiation, mRNA elongation, mRNA degradation, translation, and protein degradation [12–16]. In *Saccharomyces cerevisiae*, the complex consists of nine proteins, including five *Sc*Not proteins, three *Sc*Caf proteins, and one *Sc*Ccr4 protein [17, 18]. The *Sc*Not proteins (*Sc*Not1-5) are negative regulators of genes lacking a canonical TATA box [19]. The *ScCCR4* (carbon catabolite repression) gene positively regulates glucose-repressible enzymes [20]. The *Sc*Caf (*Sc*CCr4 associated factor) proteins *Sc*Caf1 (also known as *Sc*Pop2), *Sc*Caf40, and *Sc*Caf130 physically interact with *Sc*Ccr4 [21, 22]. Other proteins, including *Sc*Caf4, *Sc*Caf16, *Sc*Dhh1, and *Sc*Btt1, have also been shown to associate with the core of the Ccr4-Not complex [14]. In human cells, two genes (*CNOT7* and *CNOT8*) are orthologous to yeast *ScCAF1*, and *Sc*Ccr4 orthologs are also encoded by separated genes, *CNOT6* and *CNOT6L* [23]. In contrast, there is only one gene (*CNOT3*) ortholog for yeast *ScNOT3* and *ScNOT5*, which likely originated from a gene duplication event in yeast.

Yeast *ScNOT5* is involved in diverse cellular processes, including maintaining cell wall integrity, carbon catabolite repression, and filamentation [14, 24], and it has recently been identified as an essential cellular regulator linking transcription, mRNA degradation, and translation [25]. *CaNOT5* is important in morphogenesis and virulence [26], and deletion of *CaNOT5* affects cell wall structure and adherence properties in *Candida albicans* [27]. In humans, *CNOT3* is an important regulator of biological processes such as retinal homeostasis, heart physiology, and stem cell self-renewal [28–30].

In a previous work, a systemic functional analysis identified transcription factors (TFs) related to various developmental processes and virulence in *F*. *graminearum* [9]. *FgNOT3* (FGSG_13746) was shown to encode the *Sc*Not3 homolog, and Δ*Fgnot3* mutants showed pleiotropic defects in vegetative growth, sexual reproduction, secondary metabolite production, and virulence. We hypothesized that *Fg*Not3 is involved in diverse regulation, leading to severe impacts on numerous features of the fungus. In the present study, we report an in-depth functional analysis of *Fg*Not3, a member of the Ccr4-Not complex, in *F*. *graminearum*. Furthermore, we demonstrate how the functions of *Fg*Not3 are conserved in this fungus and elucidate the involvement of the Not module in the developmental stages of *F*. *graminearum*.

## Materials and Methods

### Fungal strains and media

The *F*. *graminearum* wild-type strain Z-3639 [31] and the mutants used in this study are listed in Table 1. For genomic DNA (gDNA) isolation, each strain was inoculated in 5 ml of complete medium (CM) and incubated at 25°C for 3 days on a rotary shaker at 150 rpm. For fungal sporulation, the conidia of all strains were induced on yeast malt agar (YMA) [32] and in carboxymethyl cellulose (CMC) medium [33]. A rice culture was used to evaluate trichothecene and zearalenone (ZEA) production [34]. Other media used in this study were prepared and used according to the instructions in the *Fusarium* laboratory manual [4]. The wild-type and transgenic strains were stored as mycelia and conidia in 20% glycerol at ˗80°C.

**Table 1:** *F*. *graminearum* strains used in this study.

Strain	Genotype	Reference, source, or parent strains
Z-3639	Wild-type	[31]
hH1-GFP	*hH1*::*hH1-GFP-HYG*	[35]
mat1g	Δ*mat1-1*::*GEN hH1*::*hH1-GFP-HYG*	[35]
Δ*Fgnot3*	Δ*Fgnot3*::*GEN*	[9]
FgNot3c	Δ*Fgnot3*::*FgNOT3-HYG*	This study
Δ*Fgnot3*-g	Δ*Fgnot3*::*GEN hH1-GFP-HYG*	This study
Δ*Fgnot2*	Δ*Fgnot2*::*GEN*	This study
FgNot2c	Δ*Fgnot2*::*FgNOT2-HYG*	This study
Δ*Fgnot4*	Δ*Fgnot4*::*GEN*	This study
FgNot4c	Δ*Fgnot4*::*FgNOT4-HYG*	This study

### Nucleic acid manipulation, primers, and PCR conditions

The gDNA was extracted as previously described [4]. Restriction endonuclease digestion, agarose gel electrophoresis, gel blotting, and DNA blot hybridization were performed in accordance with standard techniques [36]. The polymerase chain reaction (PCR) primers (S1 Table) used in this study were synthesized by an oligonucleotide synthesis facility (Bionics, Seoul, Korea).

### Genetic manipulations and fungal transformations

For complementation of the Δ*Fgnot3* deletion mutants, the wild-type *FgNOT3* allele from *F*. *graminearum* strain Z-3639 was amplified using the Not3-5F com/Not3-3N com primer pair. The hygromycin resistance cassette (*HYG*) was amplified from the pBCATPH vector using the pBCATPH/comp 5′For/pBCATPH/comp 3′Rev primer pair [37]. The resulting amplicons were fused by double-joint (DJ) PCR as previously described [38]. The final PCR constructs were obtained by nested PCR and transformed into the Δ*Fgnot3* deletion mutants as described previously [39].

To generate *FgNOT2* deletion mutants, the 5′- and 3′-flanking regions of the *FgNOT2* gene and a geneticin resistance cassette (*GEN*) were amplified from Z-3639 and pII99, respectively, and were fused by DJ PCR. The subsequent procedures for the third round of PCR and transformation were the same as for complementation using the *FgNOT3* gene of *F*. *graminearum*. The *FgNOT4* deletion mutants were produced using the same strategy. The same strategy used for the generation of FgNot3c strains was also applied for the complementation of the Δ*Fgnot2* and Δ*Fgnot4*.

### Conidial production and morphology

After each strain was incubated in 50 ml of CM for 72 h at 25°C on a rotary shaker (150 rpm), mycelia of each strain were harvested and washed twice with distilled water. To induce conidiation, harvested mycelia were spread on YMA and incubated for 48 h at 25°C under near-UV light (wavelength: 365 nm, HKiv Import & Export Co., Ltd., Xiamen, China). Conidia were collected using distilled water, filtered through cheesecloth, washed, and resuspended in distilled water. After inoculating a 1 ml conidial suspension (1 × 10^6^ conidia/ml) of each strain in 50 ml of CMC and incubating for 5 days at 25°C on a rotary shaker (150 rpm), the number of conidia produced was counted to measure conidial production with a hemocytometer (Superior, Marienfeld, Germany). For observation of conidial morphology, the conidia produced by each strain on YMA were harvested, and differential interference contrast (DIC) images were obtained using a DE/Axio Imager A1 microscope (Carl Zeiss, Oberkochen, Germany).

### Germination assay

To evaluate germination rates, conidial suspensions (1 × 10^6^ conidia/ml) of each strain were inoculated into 20 ml of CM and MM and incubated at 25°C on a rotary shaker (150 rpm). The germinated conidia per 100 total conidia were counted at 0, 4, 6, 8, 10, 12, 24, 36, 48, and 60 h after inoculation. Conidial germination was defined as the point at which the length of the germ tube is the same as the width of the conidium. The experiments were performed twice with three replicates for each time point.

### Outcrosses and virulence test

For self-fertilization, mycelia grown on carrot agar for 5 days were mock-fertilized with a 2.5% Tween 60 solution to induce sexual reproduction as previously described [4]. For outcrosses, mycelia of the female strain grown on carrot agar plates were fertilized with 1 ml of male strain conidia (1 × 10^6^ conidia/ml). The heterothallic mat1g (Δ*mat1-1*::*GEN hH1*::*hH1-GFP-HYG*) strain [35] was used as a tester strain for outcrosses. After sexual induction, the fertilized cultures were incubated for 7 days under near-UV light (HKiv Import & Export Co., Ltd.) at 25°C.

A virulence test of the fungal strains was performed using the wheat cultivar Eunpamil as previously described [40]. Briefly, 10 μl of conidial suspensions (1 × 10^6^ conidia/ml) obtained from each strain was point-inoculated into a spikelet of the wheat head at early anthesis. Infected plants were incubated in a humidity chamber for 3 days and subsequently transferred to a green house. After 21 days, the number of spikelets showing disease symptoms was counted.

### Quantification of mycotoxins and fungal ergosterol

For trichothecene analysis, the 3-week-old rice cultures were ground and extracted with an ethyl acetate/methanol mixture (4:1, v/v) as previously described [34]. The extracts were purified using MycoSep^®^ 225 Trich Multifunctional columns (Romer Labs, Inc., Union, MO, USA) and then concentrated to dryness. A portion of each extract was derivatized with Sylon BZT (BSA + TMCS + TMSI, 3:2:3, Supelco, Bellefonte, PA, USA) and analyzed using a Shimadzu QP-5000 gas chromatograph-mass spectrometer (GC-MS; Shimadzu, Kyoto, Japan). ZEA was extracted from rice cultures using the same strategy and analyzed using a HPLC system with a RF-10A XL fluorescence detector (Shimadzu) [34]. To quantify fungal ergosterol, ground rice cultures (1 g) were extracted in 4 ml of chloroform/methanol (2 :1, v/v) as previously described [41]. Ergosterol was analyzed using a HPLC system with a 4.6 U ODS column (250×4.6 mm, Phenomenex, Madrid Avenue Torrance, CA, USA) and an UV detector (Shimadzu) set to measure absorbance at 282 nm. Quantities were determined by comparing peak areas of samples to those of a standard curve generated from HPLC-grade ergosterol (Sigma-Aldrich, St. Louis, Missouri, USA). The experiments were repeated three times.

### Quantitative real-time (qRT)-PCR

The total RNA of the wild-type and Δ*Fgnot3* strains was extracted from mycelia at 18 h after inoculation in CMC using an Easy-Spin Total RNA Extraction kit (Intron Biotech, Seongnam, Korea) [42]. First-strand cDNA was synthesized with the SuperScript III First-Strand Synthesis System (Invitrogen, Carlsbad, CA, USA) using oligo(dT)_20_ according to the manufacturer’s recommendations. qRT-PCR was performed using iQ SYBR Green Master Mix (Bio-Rad, Hercules, CA, USA) and a 7500 real-time PCR system (Applied Biosystems, Foster City, CA, USA). The endogenous housekeeping gene, ubiquitin C-terminal hydrolase (*UBH*; FGSG_01231), was used for normalization [22]. The PCR assays were repeated three times with two biological replicates. The threshold cycle (Δ*C*_*T*_) value of gene expression was subtracted from the Δ*C*_*T*_ value of each sample to obtain a ΔΔ*C*_*T*_ value. The transcript level relative to the calibrator was expressed as 2^˗ ΔΔ*CT*^ [43].

### Yeast strains and complementation assay

The *S*. *cerevisiae* strains, BY4741 (wild-type) and YPR072w (Δ*Scnot5*), were obtained from EUROSCARF (http://web.uni-frankfurt.de/fb15/mikro/euroscarf/) and maintained on yeast extract peptone dextrose (YPD) medium. The *FgNOT3* ORF was amplified from first-strand cDNA of the *F*. *graminearum* wild-type strain, Z-3639, with the Not3-cloning-F/Not3-cloning-R primer pair (S1 Table) by PCR, digested by BstXI and XbaI restriction enzymes, and subsequently cloned into the BstXI and XbaI sites of pYES2 (Invitrogen). The pYES2-*FgNOT3* construct was introduced into the Δ*Scnot5* strain using the lithium acetate method [44] after verification of the construct by sequencing (Macrogen Inc., Seoul, Korea). In addition, the pYES2 empty vector was simultaneously introduced into yeast wild-type BY4741 and Δ*Scnot5*. Synthetic complete medium lacking uracil (SC-Ura) and supplemented with ampicillin (0.2 mg/ml) was used for the selection and isolation of transformants [44]. For the complementation assay, yeast cells were cultured for 3 days at 30°C on a rotary shaker (200 rpm) in SC-Ura supplemented with ampicillin, and the cells were then harvested and diluted in distilled water. Aliquots of 10 μl were point-inoculated on SC-Ura supplemented with ampicillin followed by incubation for 4 days at 30°C.

## Results

### Molecular characterization of the *FgNOT3* gene

**Fig 1:**
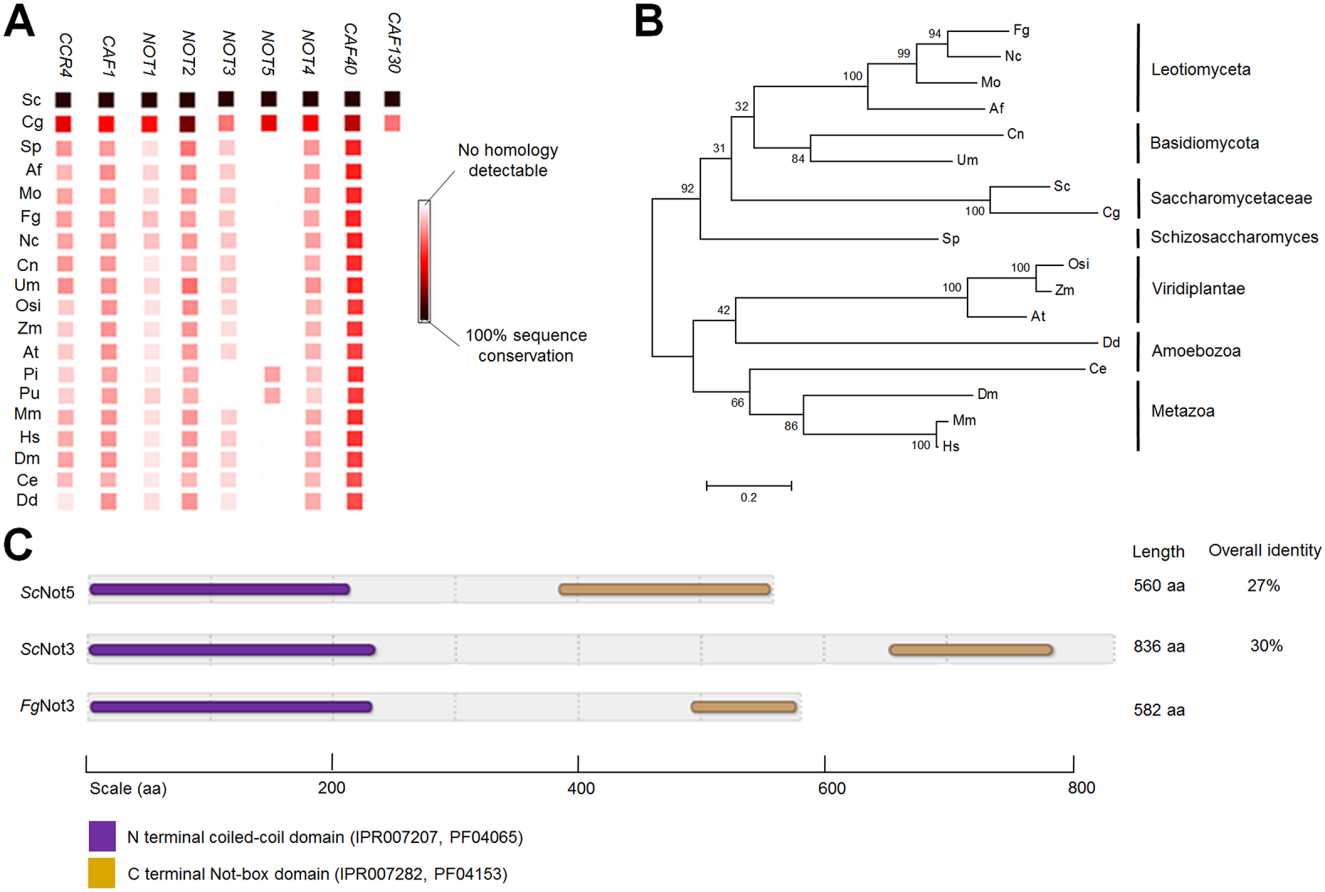
Molecular characterization of *Fg*Not3. (A) Occurrences of subunit genes of the Ccr4-Not complex homologs in representative species. The image was constructed using the STRING database [45]. Sc, *Saccharomyces cerevisiae*; Cg, *Candida glabrata*; Sp, *Schizosaccharomyces pombe*; Af, *Aspergillus fumigatus*; Mo, *Magnaporthe oryzae*; Fg, *Fusarium graminearum*; Nc, *Neurospora crassa*; Cn, *Cryptococcus neoformans*; Um, *Ustilago maydis*; Osi, *Oryza sativa Indica*; Zm, *Zea mays*; At, *Arabidopsis thaliana*; Pi, *Phytophthora infestans*; Pu, *Pythium ultimum*; Mm, *Mus musculus*; Hs, *Homo sapiens*; Dm, *Drosophila melanogaster*; Ce, *Caenorhabditis elegans*; Dd, *Dictyostelium discoideum*. (B) Phylogenetic tree of homologs of the Not3 subunit from the Ccr4-Not complex from representative species constructed using amino acid sequence comparison. (C) Schematic presentation of the conserved regions of Not3 subunit homologs between *S*. *cerevisiae* and *F*. *graminearum*. The percentage of identity between two proteins was calculated using the ALIGN algorithm (global alignment with no short-cuts). Different shadings denote the domain entries in the InterPro database (http://www.ebi.ac.uk/interpro/) and the HMMPham database (http://pfam.sanger.ac.uk/).

The Ccr4-Not complex of *S*. *cerevisiae* consists of two major modules, the catalytic module (*Sc*Caf1 and *Sc*Ccr4) and the Not module (*Sc*Not1, *Sc*Not2, *Sc*Not3, *Sc*Not4, and *Sc*Not5), and an additional two subunits, namely *Sc*Caf40 and *Sc*Caf130 [17, 18]. The occurrence of the subunit genes in the Ccr4-Not complex in representative species based on the STRING database [45] showed that most of these genes are highly conserved in eukaryotes (Fig 1A). However, Caf130 homologs are specifically conserved in members of the Saccharomycetaceae, such as *S*. *cerevisiae* and *Candida glabrata*.

Except for fungal species of the Saccharomycetaceae, only single gene-encoding proteins similar to *Sc*Not3 or *Sc*Not5 have been identified in other eukaryotic genomes (Fig 1A). Although Not3/5 of oomycetes, *Phytophthora infestans* and *Pythium ultimum* showed higher sequence identity with *Sc*Not5 than *Sc*Not3, the rest of the single proteins were homologs for *Sc*Not3. Previous reports have also shown that Not5 is not conserved in animals and is specific for the Saccharomycetaceae [46]. Phylogenetic analyses of Not3 homologs showed that Not3 homologs in filamentous fungi were clustered into a separate group relative to yeasts and animals (Fig 1B).

BLASTp searches for both *Sc*Not3 and *Sc*Not5 in the *F*. *graminearum* genome (http://www.broadinstitute.org) identified the FGSG_13746 locus encoding 582 amino acids (30% and 27% overall identity to *Sc*Not3 and *Sc*Not5, respectively). The protein harbored two significant domains (IPR007207 and IPR007282) similar to those of both *Sc*Not3 and *Sc*Not5 (Fig 1C). Further analysis of the conserved protein sequences of partial N-terminal regions showed that FGSG_13746 shares 55% and 50% identity with those of *Sc*Not3 and *Sc*Not5, respectively. The human C-terminal region of Not3 also shows 24% overall identity to both *Sc*Not3 and *Sc*Not5 although their N-terminal regions share 41% and 39% identity with those of *Sc*Not3 and *Sc*Not5, respectively [47]. Based on these combined results, we designated the protein encoded by FGSG_13746 as *Fg*Not3.

### Effects of *FgNOT3* deletion on vegetative growth, conidiogenesis, and germination

**Fig 2:**
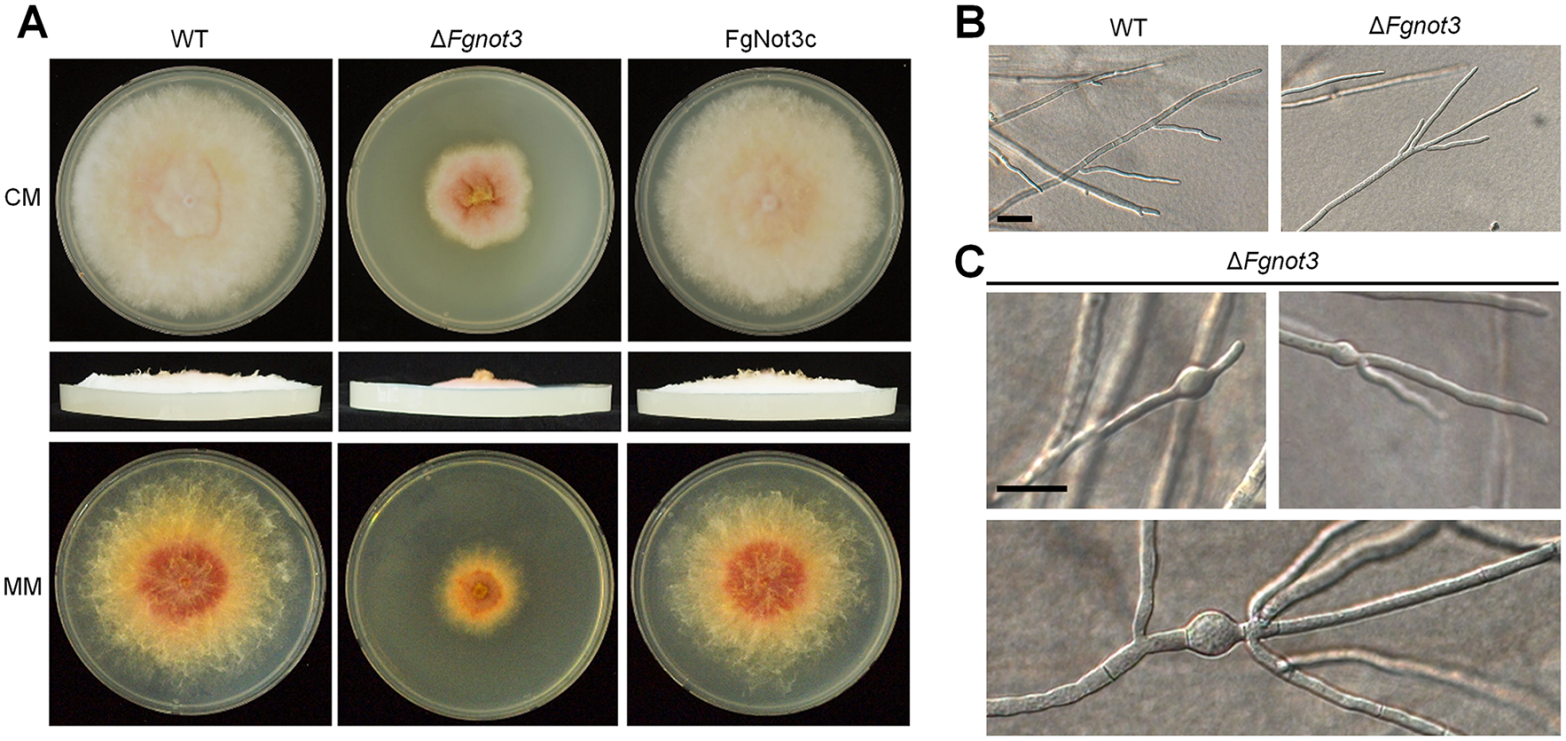
The vegetative growth of Δ*Fgnot3* mutants. (A) Mycelial growth on complete medium (CM) and minimal medium (MM). The pictures were taken 5 days after inoculation. The pictures were taken from the upper (top) and the side (middle) of the plates. (B) Microscopic observation of hyphae. The differential interference contrast (DIC) images were taken 2 days after inoculation. Scale bar = 50 μm. (C) Swollen hyphae of Δ*Fgnot3* mutants on CM agar. Scale bar = 50 μm. WT, *F*. *graminearum* wild-type strain Z-3639; Δ*Fgnot3*, *FgNOT3* deletion mutant; FgNot3c, Δ*Fgnot3*-derived strain complemented with *FgNOT3*.

The *FgNOT3* deletion mutants were obtained from a mutant library of *F*. *graminearum* TF deletions [9]. For genetic complementation, the construct containing the *FgNOT3* open reading frame (ORF) fused with *HYG* was introduced into the protoplast of the Δ*Fgnot3* strain (S1 Fig). Southern blot analysis showed that the construct successfully replaced *GEN* in the genome of the complementation strain, resulting in FgNot3c strains. The Δ*Fgnot3* strains showed markedly reduced radial growth (~50%) and aerial mycelia on both complete medium (CM) and minimal medium (MM) (Fig 2A).

To further determine the features affecting the defective growth of the Δ*Fgnot3* mutants, we performed microscopic observation. Deletion of *FgNOT3* resulted in a hyper-branching phenotype compared to the wild-type strain (Fig 2B). Furthermore, the hyphae of Δ*Fgnot3* mutants tended to be abnormally swollen, and the swollen hyphae resulted in distorted branching (Fig 2C). These results demonstrated that *FgNOT3* is required for normal growth and mycelial morphology in *F*. *graminearum*.

**Fig 3:**
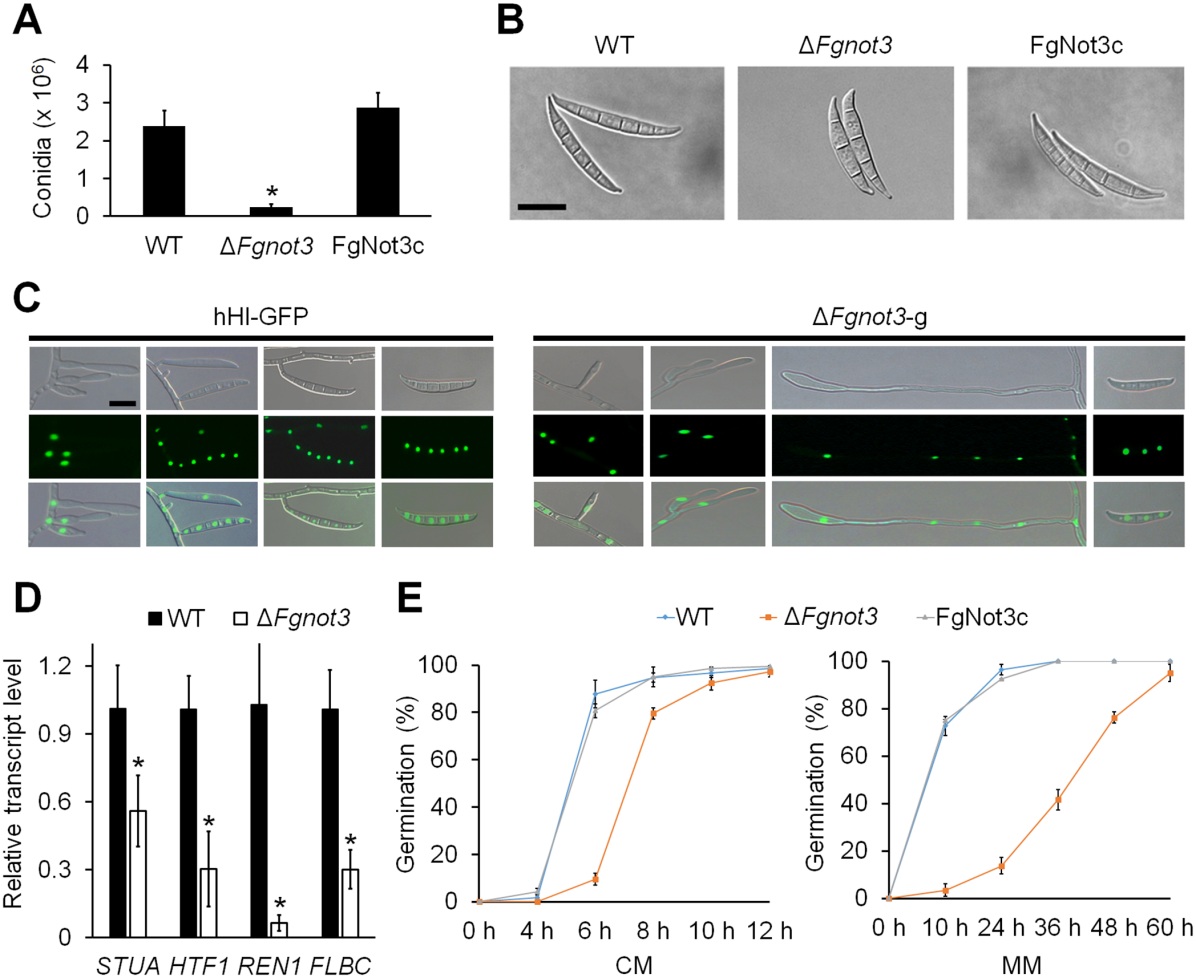
Conidiation and germination of Δ*Fgnot3* mutants. (A) Conidial production. The number of conidia was counted after 5 days of incubation in CMC. The values were generated based on three biological replicates. Significant differences (*P* < 0.05) are indicated with an asterisk. (B) Conidial morphology. Conidia were induced on YMA and subsequently observed by DIC. Scale bar = 20 μm. (C) Morphology of conidiophores in *F*. *graminearum* strains. Pictures were taken 1 to 3 days after conidium induction. Scale bar = 10 μm. (D) Relative transcript levels of genes related to conidiation. Total RNA of the wild-type and Δ*Fgnot3* strains was extracted 18 h after inoculation in CMC. The relative transcript levels of each subunit gene in the wild-type were arbitrarily set to 1. Significant differences (*P* < 0.05) are indicated with an asterisk. (E) Germination rate. The percentage of conidium germination in CM and MM. WT, *F*. *graminearum* wild-type strain Z-3639; Δ*Fgnot3*, *FgNOT3* deletion mutant; FgNot3c, Δ*Fgnot3*-derived strain complemented with *FgNOT3*.

Deletion of *FgNOT3* also resulted in severe defects in asexual sporulation. The conidial production of the Δ*Fgnot3* strain in CMC medium was significantly reduced compared to the wild-type and complemented strains (Fig 3A). Moreover, conidia of Δ*Fgnot3* strains were abnormally shaped (Fig 3B). The conidia of the Δ*Fgnot3* strains were shorter and wider than the wild-type (Table 2 and Fig 3B). Deletion of *FgNOT3* also resulted in a reduced septum number.

**Table 2:** Conidial morphology and virulence of Δ*Fgnot3* mutants.

Strain	Conidial morphology^a^			Virulence (disease index)^c^
	Length (μm)	Width (μm)	No. of septa	
**Z-3639**	46.8±1.8A^b^	6.1±0.1A	4.0±0.1A	9.7±4.1A
**Δ*Fgnot3***	41.2±0.2B	7.1±0.4B	3.4±0.1B	0.45±0.3B
**FgNot3c**	45.3±1.3A	6.2±0.1A	4.0±0.1A	9.6±2.6A

^a^ Conidia were harvested from a 1-day-old YMA culture.

^b^ The presented data are average values ± standard deviations. Values within a column with different letters are significantly different (*P* < 0.05) based on Tukey’s HSD test.

^c^ The disease index (number of diseased spikelets per wheat head) of the strains was measured 21 days after inoculation.

To determine how deletion of *FgNOT3* affects conidiogenesis in *F*. *graminearum*, we generated Δ*Fgnot3*-g strains (Δ*Fgnot3*::*GEN hH1-GFP-HYG*) by an outcross between the mat1g [35] and Δ*Fgnot3* strains. Dozens of ascospores were isolated, and their genotypes were confirmed by fluorescence microscopy and PCR. The hH1-GFP strain carrying the wild-type allele of *FgNOT3* initially produced phialides from the hyphae, and mature phialide cells continuously produced conidia (Fig 3C). Additionally, conidia were often directly produced from the hyphae. In contrast, deletion of the *FgNOT3* mostly abolished phialide production, and most conidia were directly produced from the hyphae (Fig 3C). All of these defects were restored to wild-type levels in the FgNot3c complemented strains.

To test the hypothesis that *Fg*Not3 plays a role in regulating the expression of genes related to conidiogenesis, we compared the transcript levels of representative conidiation-related genes in the wild-type and Δ*Fgnot3* deletion mutant strains [6–9]. Transcript levels of four genes, namely *STUA*, *HTF1*, *REN1*, and *FLBC*, were significantly decreased in the Δ*Fgnot3* mutants compared to wild-type (Fig 3D). Interestingly, transcript levels of *ABAA* and *WETA*, the transcription factors specifically involved in conidiogenesis in *F*. *graminearum* [10, 11], were not altered when *FgNOT3* was deleted (data not shown).

The conidia germination rates of Δ*Fgnot3* mutants were greatly reduced in both CM and MM compared to wild-type (Fig 3E). Approximately 90% of conidia in the wild-type strain germinated 6 h after inoculation in CM, whereas only approximately 10% of conidia germinated in the Δ*Fgnot3* mutants (Fig 3E). Furthermore, only approximately 16% of Δ*Fgnot3* mutant conidia germinated in MM 24 h after inoculation, whereas most wild-type conidia were germinated after 24 h. Although the germinated hyphae of all strains showed an identical morphology up to 24 h after inoculation in CM (S2 Fig), the germinated hyphae of Δ*Fgnot3* mutants exhibited swollen tips 26 h after inoculation (S2 Fig and Fig 2C). All defects of the Δ*Fgnot3* mutants were restored in the FgNot3c complemented strains.

### *FgNOT3* is important for sexual development and virulence

**Fig 4:**
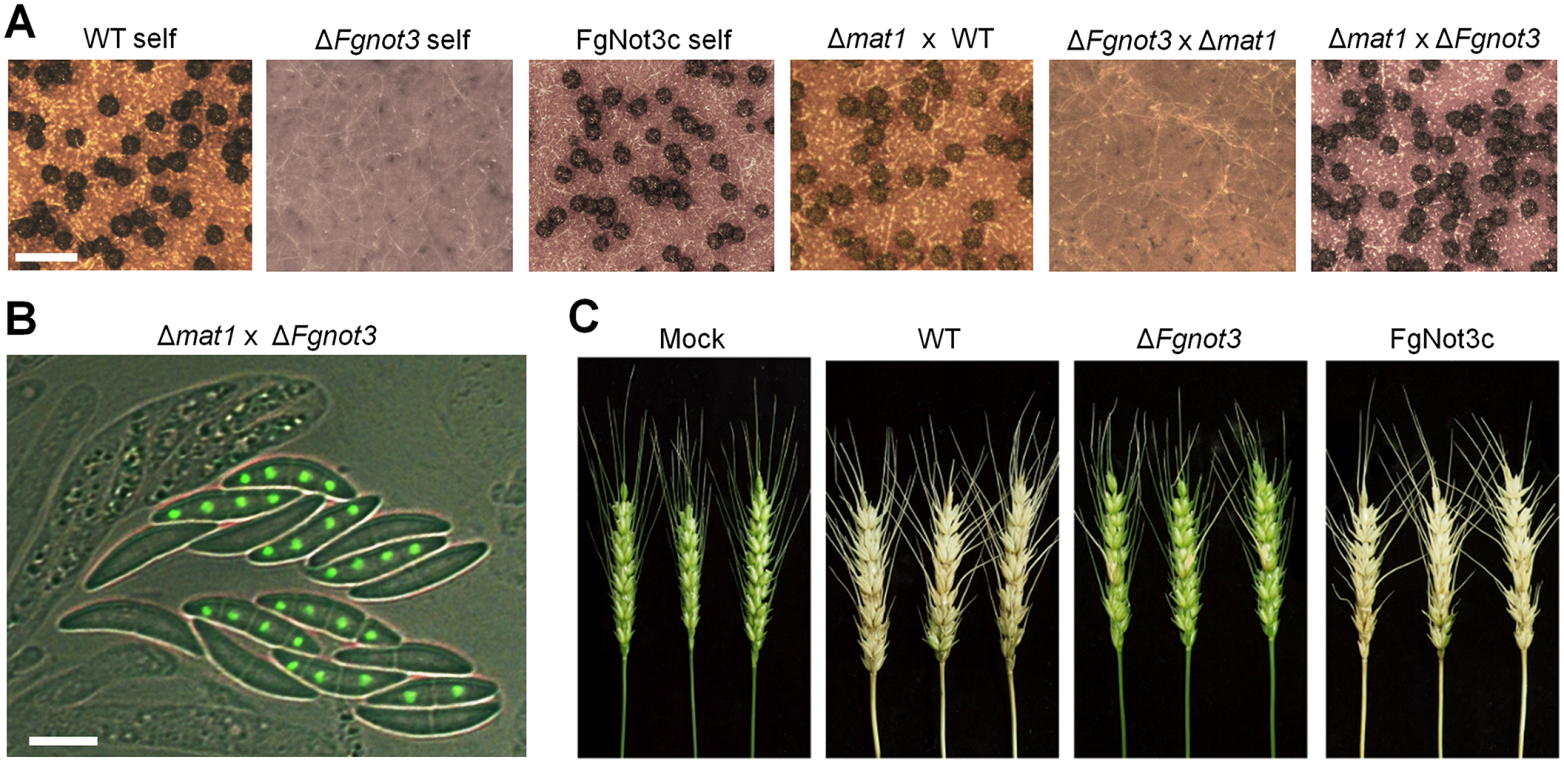
The sexual development and virulence of Δ*Fgnot3* mutants. (A) Fertility tests. Each strain was inoculated on carrot agar and mock-fertilized (self-cross) or outcrossed with the corresponding male strains (WT, Δ*Fgnot3* and Δ*mat1*). The photographs were taken 7 days after sexual induction. Scale bar = 500 μm. (B) Asci of an outcross Δ*mat1* × Δ*Fgnot3*. Eight ascospores of an ascus from the Δ*mat1* × Δ*Fgnot3* outcross showing 1:1 segregation with and without Gfp-tagged histone H1. The photographs were taken 9 days after sexual induction. Scale bar = 10 μm. (C) Virulence on wheat heads. The center spikelet of each wheat head was injected with 10 μl of a conidial suspension. Pictures were taken 21 days after inoculation. Mock, negative control mock-inoculated with 0.01% of Tween 20; WT, *F*. *graminearum* wild-type strain Z-3639; Δ*Fgnot3*, *FgNOT3* deletion mutant; FgNot3c, Δ*Fgnot3*-derived strain complemented with *FgNOT3*.

The fertility of the *F*. *graminearum* strains was determined on carrot agar. In self-fertility, the wild-type strains began to produce detectable perithecial initials 3 days after sexual induction, and mature perithecia were produced after an additional 3 or 4 days of incubation (Fig 4A). In contrast to wild-type, the Δ*Fgnot3* mutants only produced a few perithecium initials that did not mature.

Subsequently, we determined the female and male fertilities of the Δ*Fgnot3* mutants based on outcross analyses. When the Δ*Fgnot3* mutant was spermatized with wild-type or Δ*mat1* strains, no mature perithecium was produced, similar to the self-cross of the Δ*Fgnot3* mutants (Fig 4A). However, when the Δ*Fgnot3* mutant was used as a male in the outcross of Δ*mat1* (female) × Δ*Fgnot3* (male), normal perithecia were produced, and the progeny with or without the hH1-Gfp signal were segregated 1:1 in accordance with Mendelian genetics, suggesting that *FgNOT3* is not necessary for male fertility (Fig 4B). The sexual defects of the Δ*Fgnot3* mutants were recovered in the FgNot3c strains.

To evaluate the pathogenicity of the Δ*Fgnot3* mutants in flowering wheat heads, conidial suspensions of each strain were point-inoculated on spikelets. The results showed that wild-type and FgNot3c strains caused typical head blight symptoms 21 days after inoculation, whereas the Δ*Fgnot3* strains were unable to spread from the inoculated spikelet to adjacent spikelets on the heads (Table 2 and Fig 4C).

### *FgNOT3* is required for normal growth under high-temperature conditions

**Fig 5:**
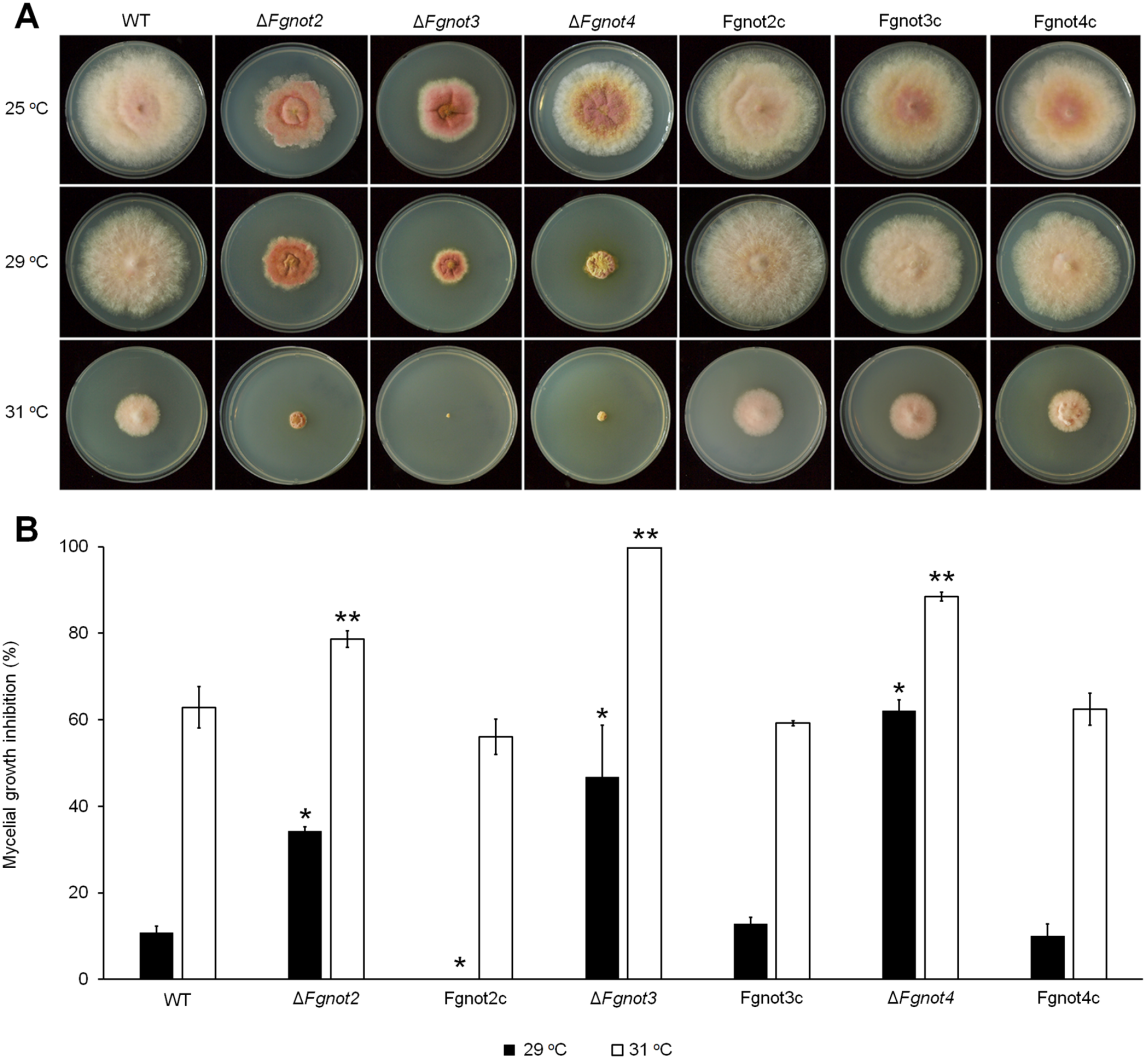
Sensitivity to thermal stress. (A) Mycelial growth at different temperatures. (B) Mycelial growth inhibition rate. Each strain was inoculated on CM and incubated at 25°C, 29°C, and 31°C for 5 days. The percentage of the mycelial radial growth inhibition was calculated using the following equation: [(C−N)/C] × 100, where C is colony diameter of the control (at 25°C), and N is that of treatments (at 29°C and 31°C) as previously described [48]. The values were generated based on three biological replicates. The significance of differences between the wild-type and each strain was calculated using Student’s t-test. Significant differences (*P* < 0.05) are indicated with an asterisk for the 29°C condition or a double asterisk for the 31°C condition. WT, *F*. *graminearum* wild-type strain Z-3639; Δ*Fgnot2*, *FgNOT2* deletion mutant; Δ*Fgnot3*, *FgNOT3* deletion mutant; Δ*Fgnot4*, *FgNOT4* deletion mutant; FgNot2c, Δ*Fgnot2*-derived strain complemented with *FgNOT2*; FgNot3c, Δ*Fgnot3*-derived strain complemented with *FgNOT3*; FgNot4c, Δ*Fgnot4*-derived strain complemented with *FgNOT4*.

To characterize the roles of *FgNOT3* in environmental stress responses, we examined the sensitivity of the Δ*Fgnot3* mutants to various stresses, including carbon and nitrogen starvation, osmotic and oxidative stresses, cell wall-damaging agents, fungicide exposure, and thermal stresses. There were no specific stresses or agents that affected the growth of Δ*Fgnot3* mutants as previously described (data not shown). However, we identified the role of *FgNOT3* in adaptation to thermal stress in *F*. *graminearum*. The Δ*Fgnot3* mutants exhibited increased sensitivity to high temperature and could not grow at 31°C (Fig 5).

### *Fg*Not3 functions together with other Not subunits of the Ccr4-Not complex

**Fig 6:**
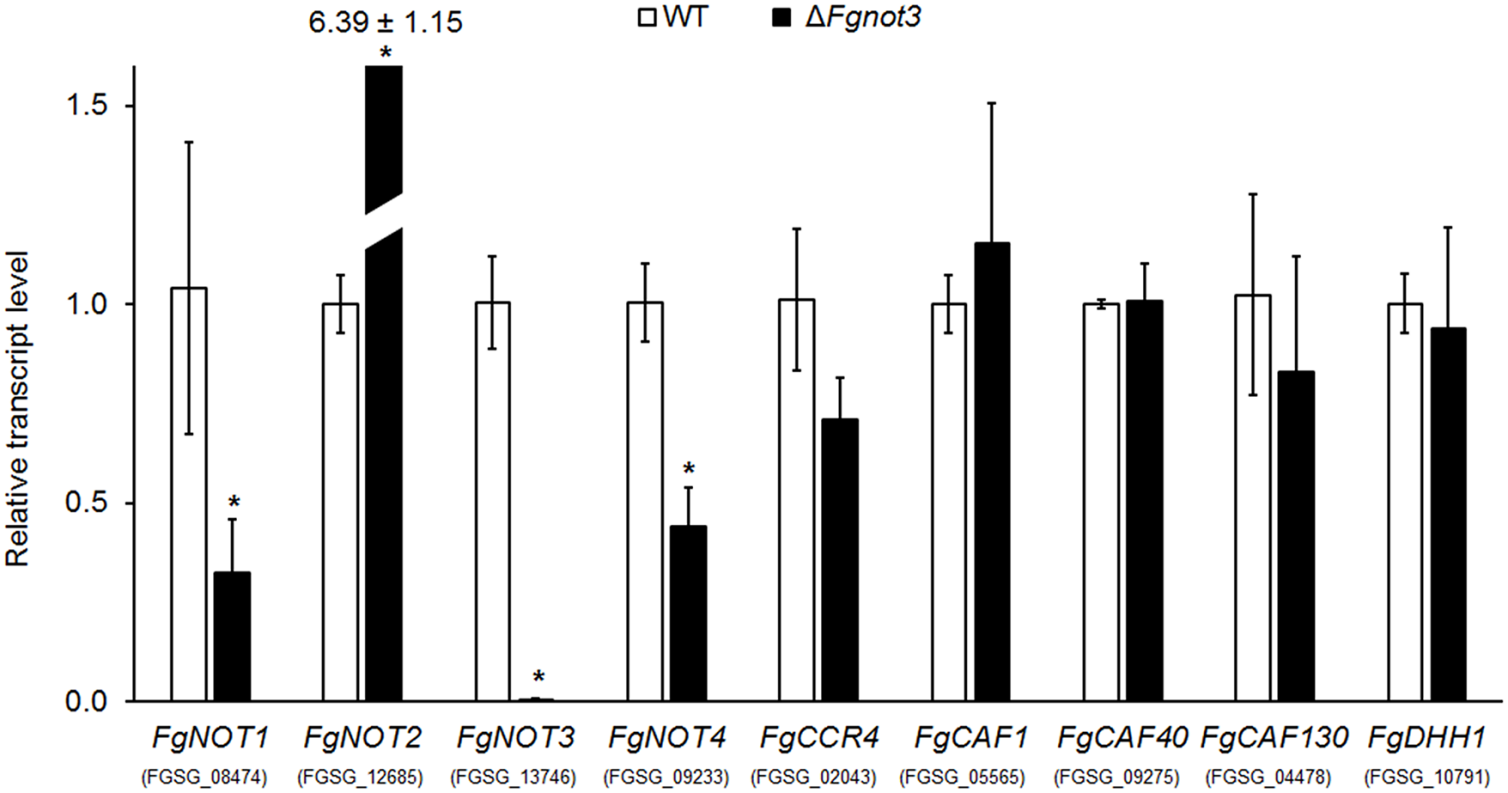
Relative transcript levels of subunits of the Ccr4-Not complex during the conidium induction stage. Total RNA of the wild-type and Δ*Fgnot3* strains was extracted 18 h after inoculation in CMC. The relative transcript levels of each subunit in the Ccr4-Not complex in wild type were arbitrarily set to 1. Significant differences (*P* < 0.05) are indicated with an asterisk.

The effects of *FgNOT3* deletion on the transcript levels of nine putative Ccr4-Not complex subunit genes were analyzed during conidiation (Fig 6). Five genes (homologs for *CCR4*, *CAF1*, *CAF40*, *CAF130*, and *DDH1*) showed similar transcript levels between the wild-type and Δ*Fgnot3* strains. However, the transcript levels of *FgNOT1* and *FgNOT4* in the Δ*Fgnot3* mutants were significantly decreased compared with the wild type (Fig 6). The *FgNOT2* transcript levels of the Δ*Fgnot3* mutants were greatly increased, showing more than 6-fold higher expression than wild type (Fig 6).

**Fig 7:**
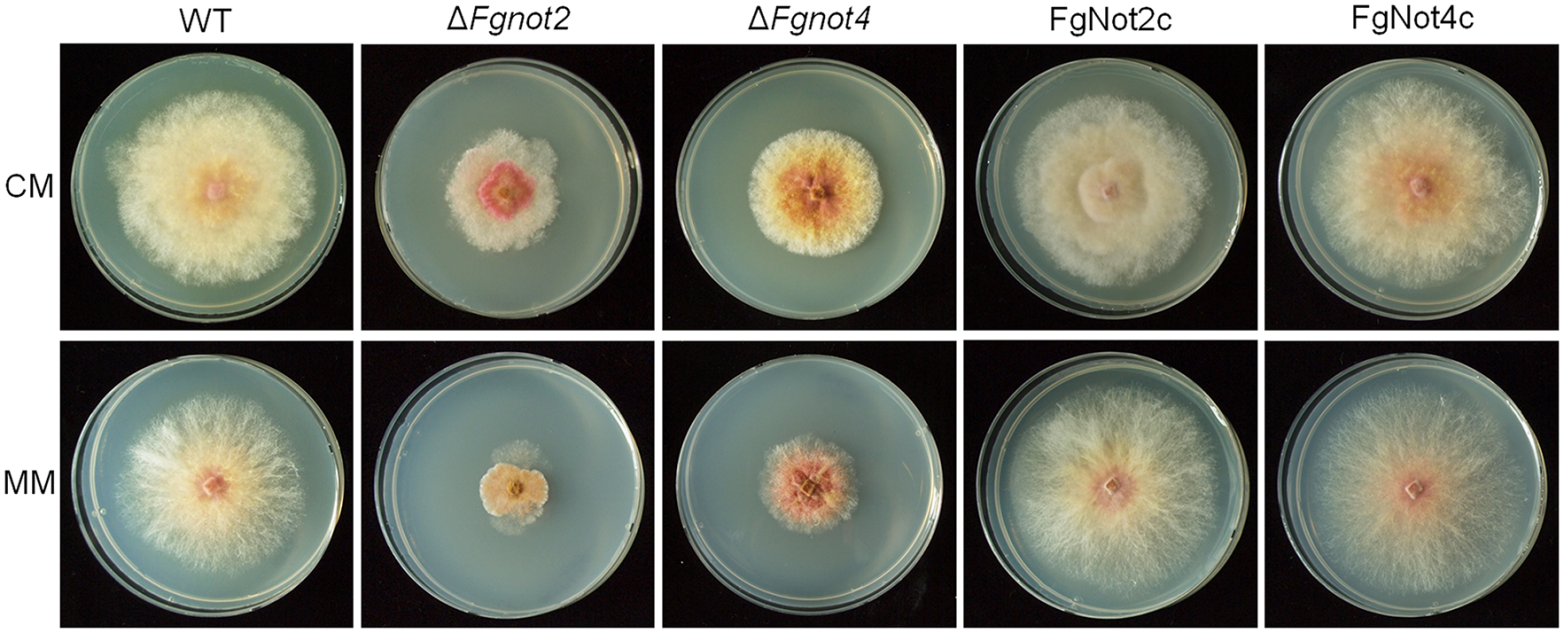
The vegetative growth of Δ*Fgnot2* and Δ*Fgnot4* mutants. Pictures were taken 4 days after inoculation on CM and MM. WT, wild-type strain Z-3639; Δ*Fgnot2*, *FgNOT2* deletion mutant; FgNot2c, Δ*Fgnot2*-derived strain complemented with *FgNOT2*; Δ*Fgnot4*, *FgNOT4* deletion mutant; FgNot4c, Δ*Fgnot4*-derived strain complemented with *FgNOT4*.

**Fig 8:**
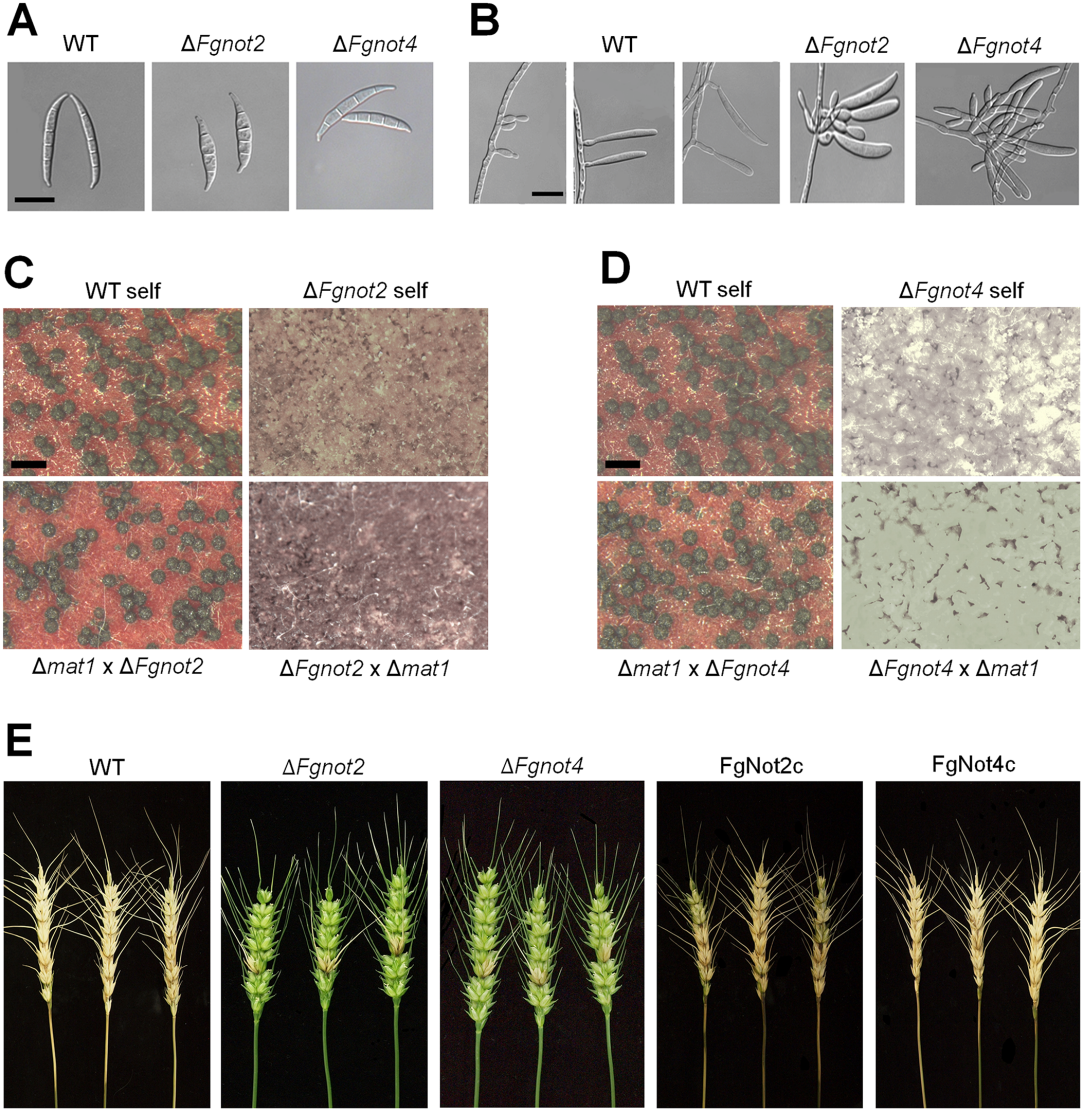
Phenotypes of Δ*Fgnot2* and Δ*Fgnot4* mutants. (A) Conidial morphology. Conidia were induced on YMA and subsequently observed by DIC. Scale bar = 20 μm. (B) Morphologies of conidiophores. Scale bar = 20 μm. (C-D) Fertility tests of Δ*Fgnot2* (C) and Δ*Fgnot4* (D). Each strain was inoculated on carrot agar and mock fertilized (self-cross) or outcrossed with a respective male strain (WT, Δ*Fgnot2*, Δ*Fgnot4*, and Δ*mat1*). Pictures were taken 7 days after sexual induction. Scale bar = 500 μm. (E) Virulence on wheat heads. The center spikelet of each wheat head was injected with 10 μl of a conidial suspension. Pictures were taken 21 days after inoculation. WT, wild-type strain Z-3639; Δ*Fgnot2*, *FgNOT2* deletion mutant; FgNot2c, Δ*Fgnot2*-derived strain complemented with *FgNOT2*; Δ*Fgnot4*, *FgNOT4* deletion mutant; FgNot4c, Δ*Fgnot4*-derived strain complemented with *FgNOT4*.

Because *FgNOT1* is an essential gene [9] and Δ*Fgnot3* showed pleiotropic defects, we sought to determine how other Not subunits affect the developmental stages of *F*. *graminearum*. To characterize their biological functions, we generated deletion and complementation mutants of *FgNOT2* (FGSG_12685) and *FgNOT4* (FGSG_09233) via homologous recombination (S4 Fig). We found that the radial growth, conidial production, conidial morphology, sexual development, and virulence of both Δ*Fgnot2* and Δ*Fgnot4* mutants were severely impaired compared to wild type, similar to the observed phenotypes of the Δ*Fgnot3* strains (Figs 7 and 8 and Table 3). In particular, deletion of *FgNOT2* and *FgNOT4* resulted in markedly reduced radial growth compared to the wild-type and complemented strains (Fig 7). Furthermore, Δ*Fgnot2* and Δ*Fgnot4* mutants also exhibited increased sensitivity to high temperature (Fig 5).

**Table 3:** Vegetative growth, conidial production, conidial morphology, and virulence of Δ*Fgnot2* and Δ*Fgnot4* mutants.

Strain	Radial growth (mm)^a^	Conidial production(10^6^/ml)^b^	Conidial morphology^c^			Virulence (disease index)^d^
			Length (μm)	Width (μm)	No. of septa	
**Z-3639**	64.4±1.1A^e^	3.1±0.5A	45.5±0.9A	5.8±0.2A	4.0±0.1A	9.8±2.3A
**Δ*Fgnot2***	50.2±1.2B	2.0±0.2B	42.9±0.2B	7.2±0.2B	3.3±0.1B	0.8±0.3B
**FgNot2c**	65.2±1.0A	2.9±0.2A	43.6±0.4B	6.1±0.1AC	4.0±0.1A	8.6±4.6A
**Δ*Fgnot4***	45.0±1.1C	2.2±0.3B	43.2±0.4B	5.9±0.1A	4.0±0.1A	2.8±2.1B
**FgNot4c**	65.6±1.6A	3.2±0.3A	46.4±1.0C	6.3±0.2C	4.2±0.2A	9.0±4.4A

^a^ Radial growth was measured 4 days after inoculation on CM plates.

^b^ Conidia were counted 5 days after inoculation in CMC.

^c^ Conidia were harvested from a 1-day-old YMA culture.

^d^ The disease index (number of diseased spikelets per wheat head) of the strains was measured 21 days after inoculation.

^e^ The presented data are average values ± standard deviations. Values within a column with different letters are significantly different (*P* < 0.05) based on Tukey’s HSD test.

Conidial production of both the Δ*Fgnot2* and Δ*Fgnot4* mutants was similarly reduced compared to wild type (Table 3). Deletion of *FgNOT2* resulted in more severe defects in conidial morphologies than *FgNOT4* deletion (Table 3 and Fig 8A). Whereas the Δ*Fgnot3* mutants showed an almost complete lack of phialide formation, the Δ*Fgnot2* and Δ*Fgnot4* mutants mainly produced normal phialides and conidia (Fig 8B). Approximately 30% of the phialides of both Δ*Fgnot2* and Δ*Fgnot4* mutants were produced as cluster forms with abnormal shapes.

We found that deletion of *FgNOT2* and *FgNOT4* also resulted in a loss of self and female fertilities (Fig 8C and 8D) and a significant decrease in virulence on wheat heads (Table 3 and Fig 8E). All of these defects of the Δ*Fgnot2* and Δ*Fgnot4* mutants were restored to wide type levels in the corresponding complemented strains.

### *FgNOT2*, *FgNOT3*, and *FgNOT4* are all involved in secondary metabolite production

**Fig 9:**
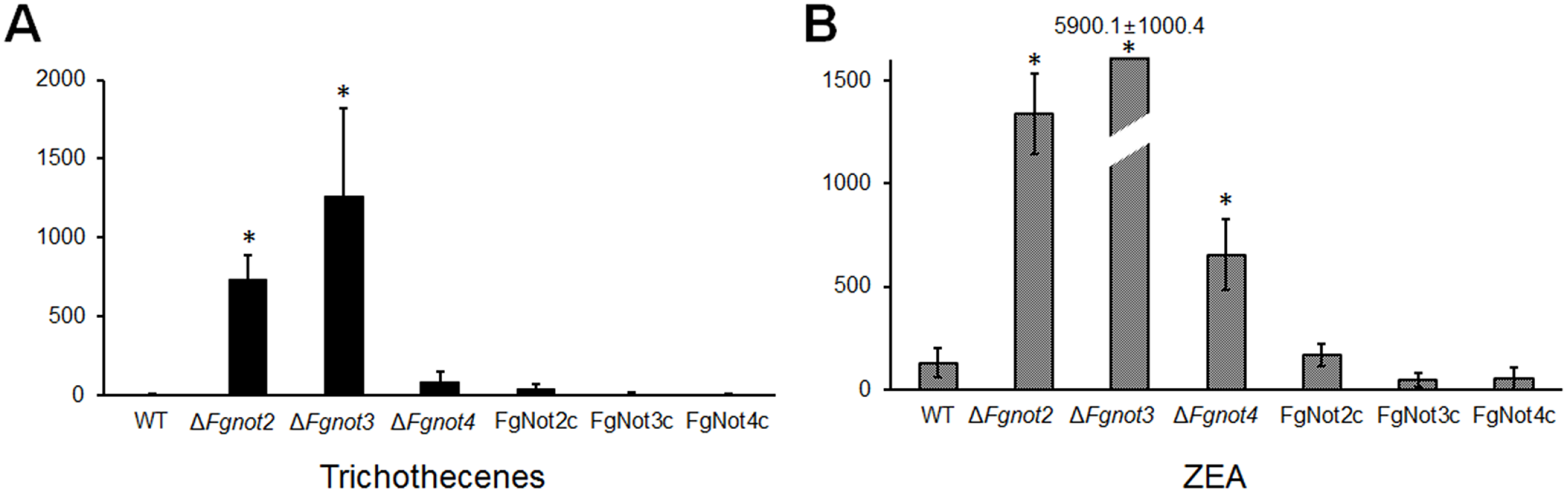
Mycotoxin production of Δ*Fgnot2*, Δ*Fgnot3*, and Δ*Fgnot4* mutants. Total trichothecene production or zearalenone (ZEA) production was normalized to fungal ergosterol levels with the following equation as previously described [41]: [trichothecene or ZEA production (μg/g) / ergosterol contents (μg/g)] × 100. The values were generated based on three biological replicates. The significance of differences between the wild-type and each strain was calculated using Student’s t-test. Significant differences (*P* < 0.05) are indicated with an asterisk. WT, wild-type strain Z-3639; Δ*Fgnot2*, *FgNOT2* deletion mutant; FgNot2c, Δ*Fgnot2*-derived strain complemented with *FgNOT2*; Δ*Fgnot3*, *FgNOT3* deletion mutant; FgNot3c, Δ*Fgnot3*-derived strain complemented with *FgNOT3*; Δ*Fgnot4*, *FgNOT4* deletion mutant; FgNot4c, Δ*Fgnot4*-derived strain complemented with *FgNOT4*.

Whereas Δ*Fgnot4* mutants only produced significantly higher levels of ZEA than the wild-type strain, deletion of both *FgNOT2* and *FgNOT3* resulted in much higher production of both trichothecenes and ZEA in rice cultures (Fig 9). These observed defects in the Δ*Fgnot2*, Δ*Fgnot3*, and Δ*Fgnot4* mutants were restored in the corresponding complemented strains.

## Discussion

In this study, *FgNOT3* was found to be involved in numerous developmental stages in *F*. *graminearum*, including vegetative growth, asexual reproduction, sexual reproduction, secondary metabolite production, and virulence. Moreover, we provided genetic evidence that other Not subunits also have conserved roles in this fungus. Taken together, these results demonstrated that the Not module of the Ccr4-Not complex plays critical roles in the regulation of multiple and complex cellular processes and differentiation in *F*. *graminearum*.

*Fg*Not3 contributes to hyphal morphogenesis and virulence in *F*. *graminearum*. Deletion of *FgNOT3* led to a significant reduction in mycelial production and abnormal shaped hyphae in *F*. *graminearum*, phenotypes similar to other model eukaryotes. Yeast *ScNOT5* is involved in diverse processes, including cell wall integrity and filamentation [14, 24]. Deletion of *CaNOT5* also results in defective morphogenesis and virulence in *Candida albicans* [26]. Although the Δ*Fgnot3* mutants produced a considerable amount of trichothecenes, which are virulence factors [49], virulence of the mutants was completely abolished, and fungal growth was restricted to infection sites. We believe that the Δ*Fgnot3* mutant hyphae could not differentiate into structures required for host infection due to the attenuated morphogenesis and cellular differentiation. It has been reported that *F*. *graminearum* hyphae develop mats and appressoria-like structures to penetrate the host cell wall [50, 51].

*Fg*Not3 also regulates hyphal differentiation required for both sexual and asexual reproduction in *F*. *graminearum*. Both ascospores and conidia are produced from highly differentiated hyphal structures such as perithecia and phialides, respectively. Δ*Fgnot3* mutants completely lost female fertility and failed to produce normal phialide cells, leading to markedly impaired spore production and abnormal conidium morphologies. The significant down-regulation of genes involved in conidiation, including *STUA*, *HTF1*, *REN1*, and *FLBC*, but not *ABAA* and *WETA*, supports the hypothesis that *FgNOT3* has global roles in asexual sporulation independent of the AbaA-WetA pathway. To our knowledge, this is the first report to implicate the contribution of Not3 homologs in both sexual and asexual developmental stages.

*NOT3/5* mutations in yeasts affect cell wall structure. Therefore, the null mutants showed altered sensitivities to cell wall stress-inducing agents. Although Δ*Canot5* mutants of *C*. *albicans* are highly sensitive to Calcofluor white, they are more resistant to β-glucanase zymolyase than wild type [26]. Δ*Scnot3* mutants show increased sensitivity to caffeine in *S*. *cerevisiae* [52]. However, *FgNOT3* deletion mutants did not show any altered sensitivity to cell wall stress-inducing agents as well as other specific stresses as previously reported (data not shown) [9]. Intriguingly, we identified a novel function of *FgNOT3* in adaptation to thermal stress. Moreover, *FgNOT3* did not complement the *S*. *cerevisiae* Δ*Scnot5* mutant (S3 Fig). Differences in the function of Not3/5 in cell wall integrity between yeast and filamentous fungi may not only be derived from evolutionary divergence between the two groups but also might explain the diverse roles of Not3/5 in cellular processes among eukaryotes. *C*Not3 in mice is essential for multiple functions, including embryonic development and control of heart function, and the metabolism of adult mice supports this notion [28, 53].

Our transcript analyses provide some clues for understanding the interactions among the subunits of Ccr4-Not complex. Transcript levels of all Not subunits, including *FgNOT1*, *FgNOT2* and *FgNOT4*, were significantly altered, but those of other Ccr4-Not subunits were not, suggesting that they function within the context of the Not module. In addition, the transcript levels of *FgNOT1* and *FgNOT4* were decreased, whereas the *FgNOT2* transcript level was highly increased in the Δ*Fgnot3* compared to wild type for unknown reasons. Because the feedback regulation often occurs in protein complexes [54], *Fg*Not2 might be a direct interactor of *Fg*Not3 but not of *Fg*Not1 and *Fg*Not4. The heterodimerization of the Not module in the ScNot1-ScNot2-ScNot5 or *C*Not1-*C*Not2-*C*Not3 forms a platform for macromolecular interactions [55, 56]. While *Sc*Not3/5 and *Sc*Not2 seem to function together [16] and *Sc*Not3 directly interacts with *Sc*Not4 and *Sc*Not5 in yeast [19, 24], there is no interaction between *C*Not3 and *C*Not4 in humans [47]. The interaction between these proteins in *F*. *graminearum* needs to be confirmed, but these differences might be attributed to a different composition of the complex or the evolutionary divergence in yeast, filamentous fungi, and human.

We further functionally characterized the Not module of the Ccr4-Not complex in *F*. *graminearum*. *FgNOT1* is an essential gene as reported in other eukaryotes [9]. Δ*Fgnot2* and Δ*Fgnot4* mutants had pleiotropic effects on phenotypes, including vegetative growth, sexual and asexual production, and virulence, similar to the impacts of Δ*Fgnot3*, suggesting that the Not module composed of *Fg*Not1-4 is also conserved in *F*. *graminearum*. In yeast, the association of all Ccr4-Not subunits is essential for cell viability although it remains unclear whether each subunit functions only within the context of a complex or has distinct roles outside of the complex [16]. For example, *Sc*Not4 mainly functions in the regulation of proteasome integrity, whereas the *Sc*Not2-3/5 module has more fundamental roles. Therefore, Δ*Sc*Not2 and Δ*Sc*Not5 showed more pronounced growth defects than Δ*Sc*Not4 [16]. Consistently, *FgNOT4* deletion had a slight effect on phenotypes, but deletion of *FgNOT2* resulted in mostly indistinguishable phenotypic defects compared to Δ*Fgnot3* mutants.

*FgNOT2*, *FgNOT3*, and *FgNOT4* are negative regulators of ZEA and/or trichothecene production. Whereas Δ*Fgnot2* and Δ*Fgnot4* mutants produced significantly higher levels of ZEA and/or trichothecenes, Δ*Fgnot3* accumulated more than 40-fold higher levels of both ZEA and trichothecenes compared with wild type. The Not module of the Ccr4-Not complex appears to regulate upstream genes or transcriptional regulatory elements participating in the diverse regulation of multiple secondary metabolite biosynthetic clusters. Moreover, highly accumulated mycotoxins might affect the physiologies of the Δ*Fgnot2*, Δ*Fgnot3*, and Δ*Fgnot4* mutants. Although direct biological functions of mycotoxins have not been reported in *F*. *graminearum*, overproduction of secondary metabolites often leads to unexpected developmental defects. All of the 13 transcription factor mutants overproducing ZEA and/or trichothecenes show defective vegetative growth and/or reproduction [9], and *FgFlbA* deletion mutants accumulating both ZEA or trichothecenes at high levels also show pleiotropic defects in *F*. *graminearum* [57].

In summary, our study functionally characterized the Not3 subunit of the Ccr4-Not complex for the first time in filamentous fungi. *FgNOT3* is involved in hyphal morphogenesis and cellular differentiation, which are related to sexual and asexual sporulation as well as virulence in *F*. *graminearum*. In addition, we found that the Not module of the Ccr4-Not complex of *F*. *graminearum* is conserved and involved in numerous characteristics, including vegetative growth, reproduction, virulence, and secondary metabolism.

## Supporting Information

S1 Fig Targeted deletion and complementation of Δ*Fgnot3*. (A) Strategies used for the deletion and complementation of Δ*Fgnot3*. The 5′-flanking regions (black bars) of the *FgNOT3* ORF were used as probes for hybridization. WT, wild-type strain Z-3639; Δ*Fgnot3*, *FgNOT3* deletion mutant; FgNot3c, Δ*Fgnot3*-derived strain complemented with *FgNOT3*; H, HindIII; P, PstI; *GEN*, geneticin resistance gene cassette; *HYG*, hygromycin B resistance gene cassette. (B) Southern blot analysis of the deletion and complementation of Δ*Fgnot3*. Lane 1, wild-type strain Z-3639; lane 2, deletion mutant; lane 3, complementation strain. The sizes of DNA standards (kb) are indicated to the left of the blot.
(TIF)

S2 Fig The mycelial morphology of Δ*Fgnot3* mutants on CM liquid medium. The mycelial morphology was observed on CM liquid medium after incubating for 8, 18, and 26 h. WT, wild-type strain Z-3639; Δ*Fgnot3*, *FgNOT3* deletion mutant; FgNot3c, Δ*Fgnot3*-derived strain complemented with *FgNOT3*.
(TIF)

S3 Fig Complementation assay of *FgNOT3* on *S*. *cerevisiae* Δ*Scnot5*. Cells were cultured for 3 days at 30°C at 200 rpm in SC lacking Ura (SC-Ura) and supplemented with ampicillin medium, harvested, and then diluted in distilled water. Aliquots of 10 μl were point-inoculated on SC-Ura supplemented with ampicillin medium and incubated for 4 days at 30°C. Columns in each panel represent serial log dilutions. BY4741, *S*. *cerevisiae* wild-type strains BY4741 harboring plasmid pYES2; Δ*Scnot5*, *S*. *cerevisiae* deletion of *Scnot5* mutant harboring plasmid pYES2; Δ*Scnot5*::*FgNOT3*, *S*. *cerevisiae* Δ*Scnot5*-derived strain complemented with *F*. *graminearum FgNOT3*.
(TIF)

S4 Fig Targeted deletion and mutant complementation strategies for *FgNOT2* (A) and *FgNOT4* (B). The 5′-flanking regions (black bars) of *FgNOT2* ORF and *FgNOT4* ORF were used as probes for hybridization. WT, wild-type strain Z-3639; Δ*Fgnot2*, *FgNOT2* deletion mutant; FgNot2c, Δ*Fgnot2*-derived strain complemented with *FgNOT2*; Δ*Fgnot4*, *FgNOT4* deletion mutant; FgNot4c, Δ*Fgnot4*-derived strain complemented with *FgNOT4*; S, SacI; *GEN*, geneticin resistance gene cassette. Lane 1, wild-type strain Z-3639; lanes 2 and 3, deletion mutants; lanes 4 and 5, complementation strains. The sizes of DNA standards (kb) are indicated to the left of the blot.
(TIF)

S1 Table Primers used in this study. (PDF)
