# Supplementary figures and images for: The FgNot3 Subunit of the Ccr4-Not Complex Regulates Vegetative Growth, Sporulation, and Virulence in Fusarium graminearum
Source: PLoS One. 2016 Jan 22;11(1):e0147481. doi: 10.1371/journal.pone.0147481 (PMC4723064; doi:10.1371/journal.pone.0147481)

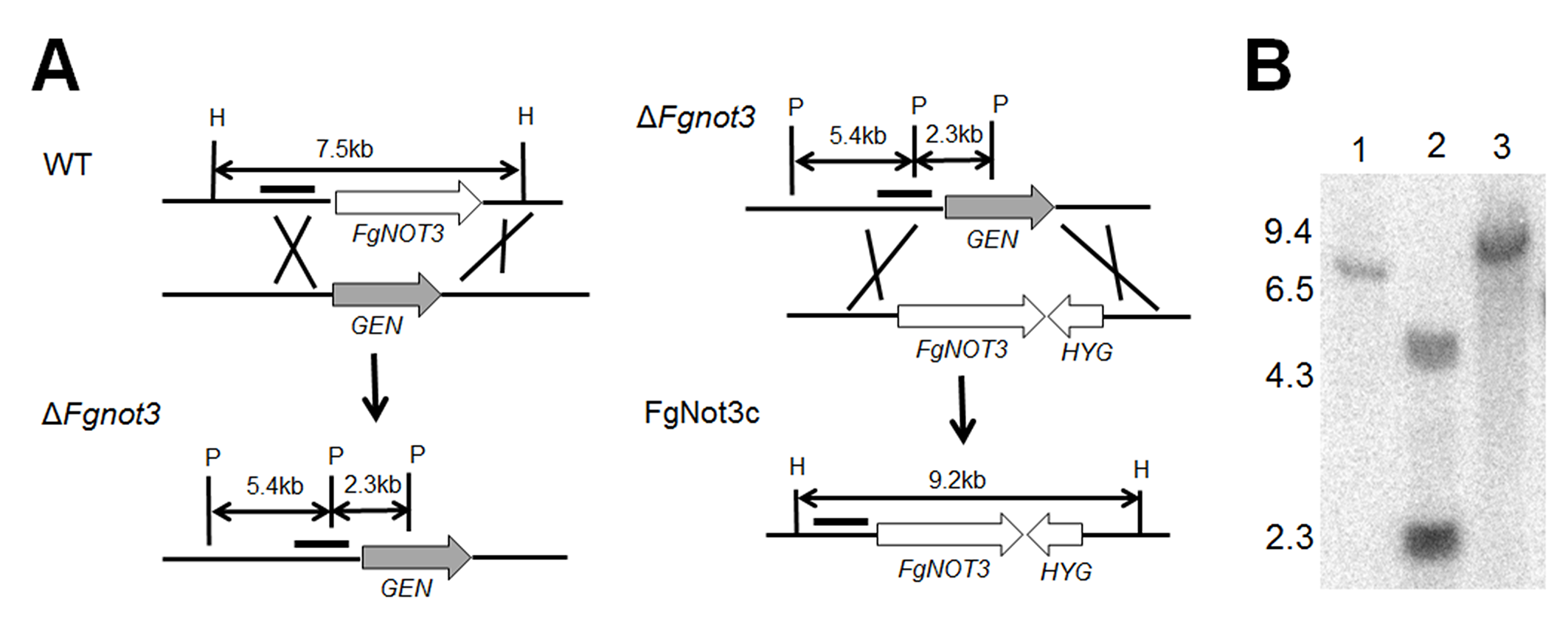

Supplement: S1 Fig — (A) Strategies used for the deletion and complementation of ΔFgnot3. The 5′-flanking regions (black bars) of the FgNOT3 ORF were used as probes for hybridization. WT, wild-type strain Z-3639; ΔFgnot3, FgNOT3 deletion mutant; FgNot3c, ΔFgnot3-derived strain complemented with FgNOT3; H, HindIII; P, PstI; GEN, geneticin resistance gene cassette; HYG, hygromycin B resistance gene cassette. (B) Southern blot analysis of the deletion and complementation of ΔFgnot3. Lane 1, wild-type strain Z-3639; lane 2, deletion mutant; lane 3, complementation strain. The sizes of DNA standards (kb) are indicated to the left of the blot. (TIF) [file pone.0147481.s001.tif]

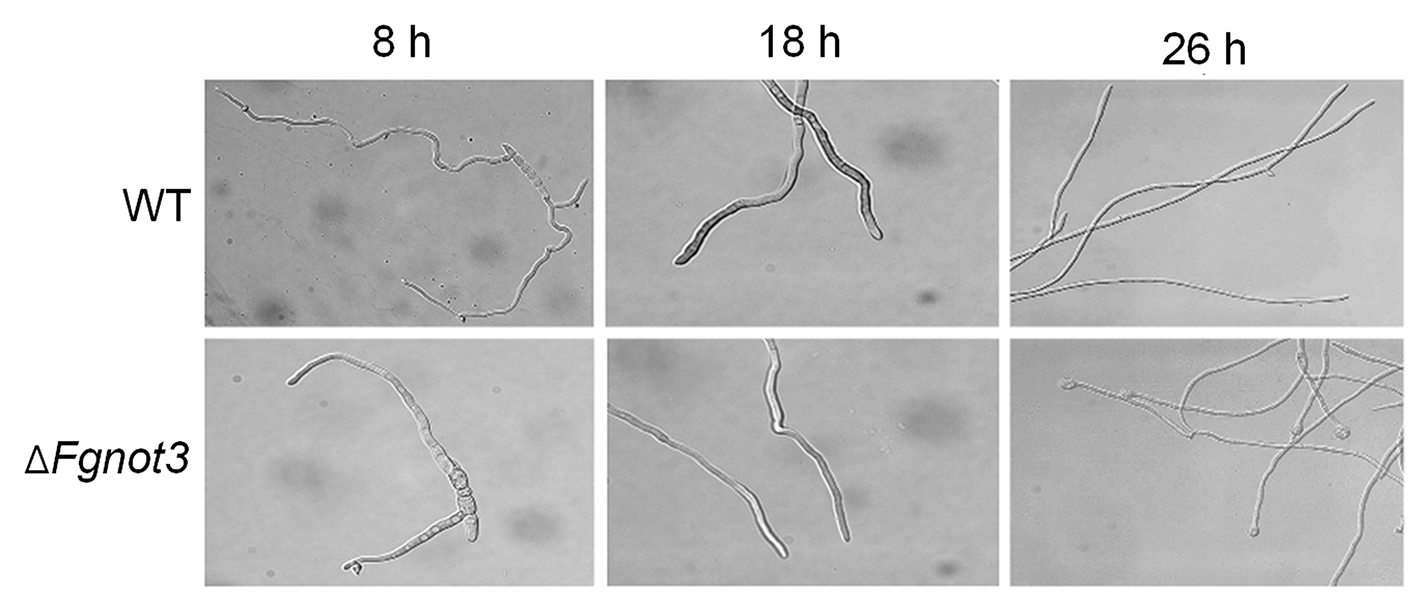

Supplement: S2 Fig — The mycelial morphology was observed on CM liquid medium after incubating for 8, 18, and 26 h. WT, wild-type strain Z-3639; ΔFgnot3, FgNOT3 deletion mutant; FgNot3c, ΔFgnot3-derived strain complemented with FgNOT3. (TIF) [file pone.0147481.s002.tif]

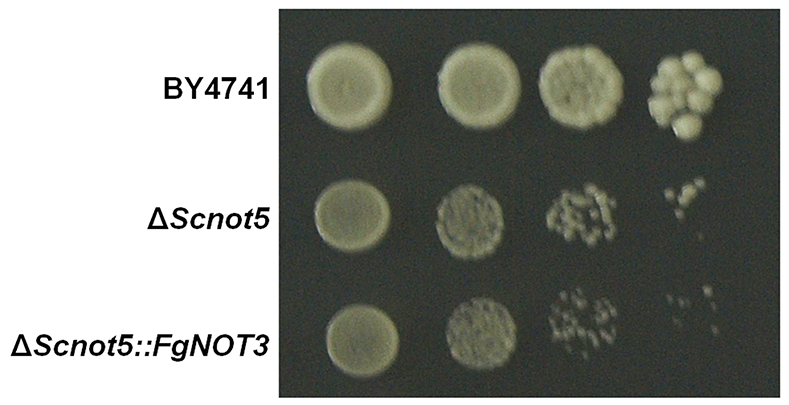

Supplement: S3 Fig — Cells were cultured for 3 days at 30°C at 200 rpm in SC lacking Ura (SC-Ura) and supplemented with ampicillin medium, harvested, and then diluted in distilled water. Aliquots of 10 μl were point-inoculated on SC-Ura supplemented with ampicillin medium and incubated for 4 days at 30°C. Columns in each panel represent serial log dilutions. BY4741, S. cerevisiae wild-type strains BY4741 harboring plasmid pYES2; ΔScnot5, S. cerevisiae deletion of Scnot5 mutant harboring plasmid pYES2; ΔScnot5::FgNOT3, S. cerevisiae ΔScnot5-derived strain complemented with F. graminearum FgNOT3. (TIF) [file pone.0147481.s003.tif]

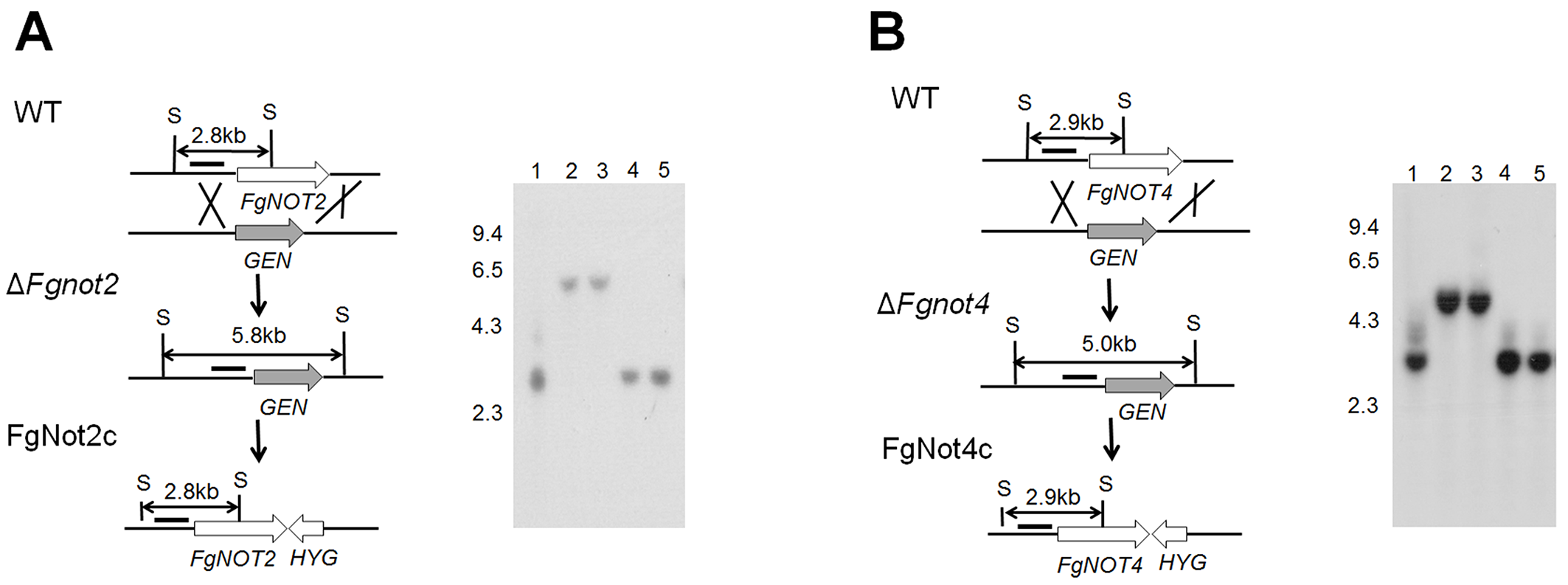

Supplement: S4 Fig — The 5′-flanking regions (black bars) of FgNOT2 ORF and FgNOT4 ORF were used as probes for hybridization. WT, wild-type strain Z-3639; ΔFgnot2, FgNOT2 deletion mutant; FgNot2c, ΔFgnot2-derived strain complemented with FgNOT2; ΔFgnot4, FgNOT4 deletion mutant; FgNot4c, ΔFgnot4-derived strain complemented with FgNOT4; S, SacI; GEN, geneticin resistance gene cassette. Lane 1, wild-type strain Z-3639; lanes 2 and 3, deletion mutants; lanes 4 and 5, complementation strains. The sizes of DNA standards (kb) are indicated to the left of the blot. (TIF) [file pone.0147481.s004.tif]
