# Supplementary material for: The FgNot3 Subunit of the Ccr4-Not Complex Regulates Vegetative Growth, Sporulation, and Virulence in Fusarium graminearum
Source: PLoS One. 2016 Jan 22;11(1):e0147481. doi: 10.1371/journal.pone.0147481 (PMC4723064; doi:10.1371/journal.pone.0147481)
Supplement: S1 Table — (PDF) [file pone.0147481.s005.pdf]

S1 Table. Primers used in this study.

Primer	Sequence (5’→3’)	Description
Not3-5F	ATCCTCAAGACCTTGGCGGC	Forward and reverse primers for amplification of 5′-flanking region of *FgNOT3* with tail for the geneticin resistance gene cassette fusion
Not3-5R	gcacaggtacacttgtttagagGCGGTTGCTAAAGACAGACAGAG	
Not3-3F	ccttcaatatcatcttctgtcgTACGAACCCTTCGAGTGCCG	Forward and reverse primers for amplification of 3′-flanking region of *FgNOT3* with tail for the geneticin resistance gene cassette fusion
Not3-3R	GGCGGCTGACGAATGCTAAC	
Not3-5N	ACAAATGTACCTTTTCCCAAAGCTC	Forward and reverse nest primers for third fusion PCR for amplification of the *FgNOT3* deletion construct
Not3-3N	TGCGCCTCGGTGACTGATAG	
Gen-for	CGACAGAAGATGATATTGAAGG	Forward and reverse primers for amplification of the geneticin cassette from the pII99 vector
Gen-rev	CTCTAAACAAGTGTACCTGTG	
pBCATPH/comp 5′For	GTGAGCGGATAACAATTTCACACAG	Forward and reverse primers for amplification of hygromycin B resistance gene cassette from pBCATPH
pBCATPH/comp 3′Rev	GAGATCCTGAACACCATTTGTCTCA	
pIGPAPA/H2	TCGCTCCAGTCAATGACCGC	Forward nest primers for split marker amplification of hygromycin B resistance gene cassette
pU,pBC/H3	CGTTATGTTTATCGGCACTTTGC	Reverse nest primers for split marker amplification of hygromycin B resistance gene cassette
Gen-G2	GCAATATCACGGGTAGCCAACG	Forward nest primers for split marker amplification of geneticin resistance gene cassette
Gen-G3	GGGAAGGGACTGGCTGCTATTG	Reverse nest primers for split marker amplification of geneticin resistance gene cassette
pIGPAPA-sGFP	GTGAGCAAGGGCGAGGAGCTG	Forward and reverse primers for amplification of

Hyg-F1	GGCTTGGCTGGAGCTAGTGGAGG	*GFP-HYG* construct from pIGPAPA
Not3-5F com	GAAAAGGGAGTCCAACAGGCAATAA	Forward primer for amplification of *FgNOT3* with tail for the hygromycin B resistance gene cassette fusion
Not3-5N com	GAAAAACCACGTCATTGAAACTATCCC	Forward nest for amplification of *FgNOT3* with tail for the hygromycin B resistance gene cassette fusion
Not3- 3N com	tgagacaaatggtgttcaggatctcGAGGCTGCAGCAAGAAAAAGAGGT	Reverse primer for amplification of *FgNOT3* with tail for the hygromycin B resistance gene cassette fusion
STUA-rt-F	CAGAACGGAAATGATGGTGGACTC	For realtime-PCR of *STUA*
STUA-rt-R	ATTGGAAAGAGGCTGGTGAAGGT	
HTF1-rt-F	GGAAGAAGAGCTGAGGTGGGACAT	For realtime-PCR of *HTF1*
HTF1-rt-R	TGGAAGTTGGGGGAGCGGT	
REN1-rt-F	ACGACAGACTTGAATCGCCTGACA	For realtime-PCR of *REN1*
REN1-rt-R	TATCGTGCCACATCGTATCCAGCA	
FLBC-rt-F	TTCAGCTCCAAGGTGTCTTCCAGT	For realtime-PCR of *FLBC*
FLBC-rt-R	ACAGAGAAATGTCGACCACAGCCT	
ABAA-rt-F	ACTCAGGAAGCTTTGACCACGGC	For realtime-PCR of *ABAA*
ABAA-rt-R	GGGCTCTGGTAGGGGTTGACAGTA	
WETA-rt-F	GTTCCAGGTACTCCCACTGCCAT	For realtime-PCR of *WETA*
WETA-rt-R	ACGTTCTCGTCGCGCTTTGGT	
CAF130-rt-F	TCATTAGACGTTCGACCCCATTTC	For realtime-PCR of *CAF130*

CAF130-rt-R	TCGGGTTGGTGATGTAGATGGTG	
CAF1-rt-F	TACTGAGTTTCCGGGTGTCGTTTC	For realtime-PCR of *CAF1*
CAF1-rt-R	AAACTGCCACGAACAAGGGAAAG	
NOT1-rt-F	TCAGCCAAACCTCCCAACAAGA	For realtime-PCR of *NOT1*
NOT1-rt-R	CAAGGAGGCATAGGCACTGACAA	
NOT4-rt-F	CCGTGCCAACATCCAGAAGAAC	For realtime-PCR of *NOT4*
NOT4-rt-R	CTCGGGCTTTCGCAGTGTCTT	
CAF40-rt-F	TCTACCCCTTTCTCAACACCACCTC	For realtime-PCR of *CAF40*
CAF40-rt-R	CCATTGTCGTCGAGCAGGATTT	
DHH1-rt-F	CGCCTTTGTCATCCCCACCT	For realtime-PCR of *DHH1*
DHH1-rt-R	ATTCGCTAAGGTCGGCAACATTC	
NOT2-rt-F	GTCCAACGCCAACCAGTCATCTAT	For realtime-PCR of *NOT2*
NOT2-rt-R	GAGGTTTGGTTGAGTGGTGGGA	
NOT3-rt-F	GATCCCAAAGAGCAGGCAAAGG	For realtime-PCR of *NOT3*
NOT3-rt-R	TGCCTTGATGCCACTTATGTCGT	
CCR4-rt-F	CACCCCTCTCAGACACAACACCA	For realtime-PCR of *CRR4*
CCR4-rt-R	GCATGCGCTCGTTCGGACT	
UBH-rt-F	GTTCTCGAGGCCAGCAAAAAGTCA	For realtime-PCR of *UBH*
UBH-rt-R	CGAATCGCCGTTAGGGGTGTCTG	
Not3-cloning-F	GCATGACCAGTGTGCTGGATGGCGGCAAGGAAACTG GCCC	Forward and reverse primer for amplification of cDNA of *FgNOT3*
Not3-cloning-R	GCATGATCTAGATAAAAAGATGGGGGAACATGTTTGA	

	TACTGC	
Not3-cloning seq-F	CGGACTACTAGCAGCTGTAATACGACTC	Forward and reverse primer for amplification of sequencing colonies containing plasmid construct pYES2-*FgNOT3*
Not3-cloning seq-R	GGTTGTCTAACTCCTTCCTTTTCGGT	
Not4-5F	GACCAGCAAAGAAGACACCGTAAAG	Forward and reverse primers for amplification of 5′-flanking region of *FgNOT4* with tail for the geneticin resistance gene cassette fusion
Not4-5R	gcacaggtacacttgtttagagTGTCGTGTGATTAGTTTGAAAGGTGGTA	
Not4-3F	ccttcaatatcatcttctgtcgTTCACGTAACGACAGGGCAGAGT	Forward and reverse primers for amplification of 3′-flanking region of *FgNOT4* with tail for the geneticin resistance gene cassette fusion
Not4-3R	CAGCGAACGACCAGACCACAGT	
Not4-5N	GCAGCGGTGTTGTCTATCAGGTTT	Forward and reverse nest primers for third fusion PCR for amplification of the *FgNOT4* deletion construct
Not4/3N	AAAAGCACAACCAAAACCCAAGAAC	
Not4-5F com	CCATCACCCAGGCACCGTATC	Forward primer for amplification of *FgNOT4* with tail for the hygromycin B resistance gene cassette fusion
Not4-5N com	GAGAATCTTAGCGGCAGAACAGTGG	Forward nest for amplification of *FgNOT4* with tail for the hygromycin B resistance gene cassette fusion
Not4-3N com	tgagacaaatggtgttcaggatctcCTCTAACATTGATGCTATCGCTTCGT	Reverse primer for amplification of *FgNOT4* with tail for the hygromycin B resistance gene cassette fusion
Not2-5F	AAGTAGAGGAGTGGTTGGAAGTTGGTAA	Forward and reverse primers for amplification of 5′-flanking region of *FgNOT2* with tail for the geneticin resistance gene cassette fusion
Not2-5R	gcacaggtacacttgtttagagCGCGAGCGACTAAGGTAAGGTG	
Not2-3F	ccttcaatatcatcttctgtcgTTAGGTGAGGGAGCATGGTGATTG	Forward and reverse primers for amplification of 3′-flanking region of *FgNOT2* with tail for the
Not2-3R	CAGGGGCCGTTATTGTTGTTCAG	

		geneticin resistance gene cassette fusion
Not2-5N	AACGGAACTCAACCAAGTCAAGGA	Forward and reverse nest primers for third fusion PCR for amplification of the *FgNOT2* deletion construct
Not2-3N	GCGATACGGCATCTAGCTTGTTCT	
Not2-5F com	CGACGCGAAAGAGGAGAGAAAT	Forward primer for amplification of *FgNOT2* with tail for the hygromycin B resistance gene cassette fusion
Not2-5N com	GTTGGACCTTTTCCCCGTGG	Forward nest for amplification of *FgNOT2* with tail for the hygromycin B resistance gene cassette fusion
Not2-3N com	tgagacaaatggtgttcaggatctcCGACAAGCCAGCCCTCAGTAAT	Reverse primer for amplification of *FgNOT2* with tail for the hygromycin B resistance gene cassette fusion
